# MECOM/PRDM3 and PRDM16 Serve as Prognostic-Related Biomarkers and Are Correlated With Immune Cell Infiltration in Lung Adenocarcinoma

**DOI:** 10.3389/fonc.2022.772686

**Published:** 2022-01-31

**Authors:** Meng Li, Hui Ren, Yanpeng Zhang, Na Liu, Meng Fan, Ke Wang, Tian Yang, Mingwei Chen, Puyu Shi

**Affiliations:** ^1^ Department of Respiratory and Critical Care Medicine, the First Affiliated Hospital of Xi’an Jiaotong University, Xi’an, China; ^2^ Department of Talent Highland, the First Affiliated Hospital of Xi’an Jiaotong University, Xi’an, China; ^3^ Department of Thoracic Surgery, the First Affiliated Hospital of Xi’an Jiaotong University, Xi’an, China; ^4^ Department of Center for Translational Medicine, the First Affiliated Hospital of Xi’an Jiaotong University, Xi’an, China; ^5^ Department of Medical Oncology, the First Affiliated Hospital of Xi’an Jiaotong University, Xi’an, China

**Keywords:** MECOM, PRDM3, PRDM16, prognosis, immune infiltration, lung adenocarcinoma

## Abstract

**Background:**

The MDS1 and EVI1 complex locus (MECOM, also called PRDM3) and PR domain containing 16 (PRDM16) are two highly related zinc finger transcription factors associated with many malignancies. However, the mechanisms of MECOM and PRDM16 in prognosis and tumor immune infiltration in lung adenocarcinoma (LUAD) remain uncertain.

**Methods:**

The Cancer Genome Atlas (TCGA), Oncomine, UALCAN, GEPIA, and TIMER databases were searched to determine the relationship between the expression of MECOM and PRDM16, clinicopathological features, immune infiltration, and prognosis in LUAD. Coexpressed genes of the two genes were investigated by CBioPortal, and the potential mechanism of MECOM- and PRDM16-related genes was elucidated by GO and KEGG analyses. STRING database was utilized to further construct the protein-protein interaction network of the coexpressed genes, and the hub genes were identified by Cytoscape. Finally, qRT-PCR was performed to identify the mRNA levels of the target genes in LUAD.

**Results:**

mRNA levels of MECOM and PRDM16 were downregulated in LUAD (*p* < 0.05), and the low expression of the two genes was associated with the age, gender, smoking duration, tissue subtype, poor stage, nodal metastasis status, TP53 mutation, and prognosis in LUAD (*p* < 0.05). MECOM and PRDM16 were also found to be correlated with the expression of a variety of immune cell subsets and their markers. KEGG analysis showed that both of them were mainly enriched in the cell cycle, cellular senescence, DNA replication, and p53 signaling pathway. Importantly, the mRNA levels of the two genes were also found to be decreased in the clinical samples of LUAD by qRT-PCR.

**Conclusion:**

MECOM and PRDM16 may serve as potential prognostic biomarkers which govern immune cell recruitment to LUAD.

## Introduction

Lung cancer, a malignant disease, poses a serious threat to human health, especially in East Asia (1). Non-small cell lung cancer (NSCLC) accounts for approximately 85% of all lung carcinoma (2), and adenocarcinoma is the most prevalent subtype (3). In recent years, significant advances have been made in targeted therapy and immunotherapy in lung cancer treatment, some of which have replaced traditional chemoradiotherapy as the first-line treatment (4). Despite the advances in lung cancer-related treatment technology, the long-time survival remains poor, with a 5-year survival rate of less than 20% (5). Therefore, it is of great significance to investigate more potential molecular targets for its diagnosis, treatment, and prognostic evaluation.

The PR/SET domain (PRDM) gene family contains 19 members, which is structurally defined by the conserved N-terminal PR domain that relates to the SET methyltransferase domain and multiple zinc fingers that mediate sequence-specific DNA binding and protein-protein interactions (6, 7). PRDM proteins are involved in a spectrum of crucial cellular processes such as cell fate determination and development, and abnormal function of some members may contribute to malignant transformation (6–8). MDS1 and EVI1 complex locus (MECOM) encodes PRDM3 (also known as MDS1-EVI1). PR domain containing 16 (PRDM16, also called MEL1) was identified as a closely related paralog of PRDM3, which shares 53% sequence identity with the N-terminus of PRDM3 (9). Given the striking similarity between MECOM/PRDM3 and PRDM16, the two are thought to have a cooperative effect on certain biological functions, the occurrence, and the development of the disease. PRDM3 and PRDM16 were found to maintain the survival of normal hematopoietic stem cells with additive roles (10), and they were also demonstrated to have a direct interaction with the NuRD chromatin remodeling complex (11). Of note, MECOM and PRDM16 are the most mutated genes in the PRDM family in multiple human cancers (12, 13), which has been verified to promote the occurrence and progression of acute myeloid leukemia (14, 15). MECOM was found to be upregulated in well-differentiated pancreatic ductal adenocarcinoma (PDAC), which contributes to better survival in PDAC patients (16). By contrast, PRDM16 was observed with extensive epigenetic gene silencing and inhibits tumor growth by suppressing HIF-targeted gene semaphorin 5B in renal cancer. (17). PRDM16 was also found to function as a suppressor in lung cancer metastasis and downregulated in LUAD (18). The potential mechanism of MECOM and PRDM16, especially MECOM, in terms of tumor progression, prognosis, and immune cell infiltration in LUAD, however, remains unclear.

Herein, we analyzed the expression of MECOM and PRDM16 in LUAD and investigate the correlation between the two genes and clinicopathological features, prognosis, immune cell infiltration, and markers of immune cells in LUAD patients on the basis of various databases. Finally, the expression levels of MECOM and PRDM16 were further verified by Quantitative reverse transcription polymerase chain reaction (qRT-PCR) in LUAD patients.

## Materials and Methods

### Analysis of MECOM and PRDM16 Expression in Different Cancers

Oncomine (19) and TIMER databases (20) were utilized to explore the mRNA levels of MECOM and PRDM16 in tumor and adjacent normal tissues. Filter criteria of Oncomine: *p* < 1E−04; multiple changes = 2. Data type was mRNA. Gene ranking is top 10%.

### Data Source and Processing

The gene expression data in HTSeq-FPKM of 535 LUAD and 59 normal lung tissues and the clinical information of 522 LUAD patients were obtained from The Cancer Genome Atlas (TCGA) database (21). A total of 57 paired LUAD and adjacent normal lung tissues among the 594 samples were identified in TCGA. Samples with incomplete parameters or lack of prognostic follow-up clinical data were excluded. The details are shown in **Table S1**.

### Expression and Survival Analysis of MECOM and PRDM16 in LUAD

The downloaded data of TCGA were analyzed by the R “limma” and “survival” package to explore the expression of MECOM and PRDM16 in LUAD, normal tissues, and different clinical features, as well as the correlation between MECOM, PRDM16, and the overall survival (OS) in LUAD. Subsequently, the association between MECOM, PRDM16, clinicopathologic features, and disease-free survival (DFS) were further analyzed by UALCAN (22) and GEPIA (23). CBioPortal database (24) is an open-platform integrated data from multiple databases including TCGA, which was searched to investigate the correlation between MECOM and PRDM16 expression and its methylation levels.

### Visualization, Expression, and Correlation Analysis of Immune Cells in LUAD

Immune cell expression in each LUAD sample of TCGA was obtained by the “CIBERSORT” R package, and the results were visualized by the “Barplot” and “Corrplot” R package. The expression of different immune cells between patients with high and low expression of MECOM and PRDM16 in LUAD were investigated by the “limma” package and visualized by the “Vioplot” package.

### Correlation Analysis Between MECOM and PRDM16 Expression, Immune Cell Infiltration, and Immune Cell Biomarkers in LUAD

The correlations between MECOM and PRDM16 and the infiltration of the immune cells in LUAD were analyzed by the Spearman correlation analysis of the R package. Gene_Corr module of the TIMER database was performed to investigate the relationship between the expression of the two genes and biomarkers of immune cells.

### Survival Analysis of MECOM and PRDM16 Expression and Immune Cell Infiltration in LUAD

The effect of gene expression and immune cell infiltration on survival of LUAD patients was investigated by the Outcome Module of the TIMER database.

### Screening of MECOM- and PRDM16-Coexpressed Genes

The coexpression genes of PRDM members MECOM and PRDM16 were analyzed using the CBioPortal database (coexpression criteria: |*r*| > 0.4 and *p* < 0.001), and heatmaps were generated using the “heatmap” package of R. The intersection genes were then extracted from the MECOM- and PRDM16-coexpressed genes by the “VennDiagram” package of R.

### GO and KEGG Analysis

The biological functions and signaling pathways affected by the coexpressed genes of MECOM and PRDM16 were investigated by GO and KEGG analyses by the “clusterProfiler” package of R (25).

### Protein-Protein Interaction Network Construction and Hub Genes Identification

STRING database was performed to construct a protein-protein interaction (PPI) network of the coexpressed genes of MECOM and PRDM16 with an interaction score of >0.7. Subsequently, the top 10 hub genes were identified using the CytoHubba plugin of Cytoscape that uses the degree method. Finally, the expression of hub genes and the relationship between them, OS, and DFS in LUAD were investigated by the GEPIA database.

### Patients and Tissue Samples

A total of 10 patients with pathologically confirmed primary LUAD in the First Affiliated Hospital of Xi’an Jiaotong University (Shaanxi, China) from July 2020 to Mar 2021 were collected in this study. All patients did not receive radiotherapy and/or chemotherapy and are without another cancer history.

### Quantitative Reverse Transcription Polymerase Chain Reaction

Total RNA was extracted from lung tissues by RNA-easyIsolation Reagent (Vazyme #R701). SuperScript IV Reverse Transcriptase (cat. no. 18090010, Thermo Fisher, Waltham, MA, USA) was performed for reverse transcription, and SYBR^®^ Premix Ex Taq™ II (Tli RNaseH Plus) (cat. no. RR820Q, Takara, Kusatsu, Japan) was utilized for the qRT-PCR in accordance with the instructions. The primer sequences are shown in **Table S2**. The thermocycling program of qRT-PCR is shown below: initial denaturation for 30 s at 95°C, 40 cycles of denaturation for 5 s at 95°C, and 40 cycles of amplification for 30 s at 60°C. The mRNA expression of MECOM and PRDM16 in cells was represented as the 2^−ΔΔCt^, and β-actin was used as the internal reference.

### Statistical Analysis

Perl, R(4.0.1), and SPSS 18.0 were used for data processing and statistical analysis. Wilcoxon test and Mann-Whitney *U* test were performed to evaluate the expression of MECOM and PRDM16 and their association with the clinicopathological features of LUAD patients. Spearman correlation analysis was conducted to assess the correlations between MECOM and PRDM16 expression and the immune infiltration in LUAD. *p* < 0.05 was statistically significant.

## Results

### The Expression of MECOM and PRDM16 Were Downregulated in LUAD

**Figure 1:**
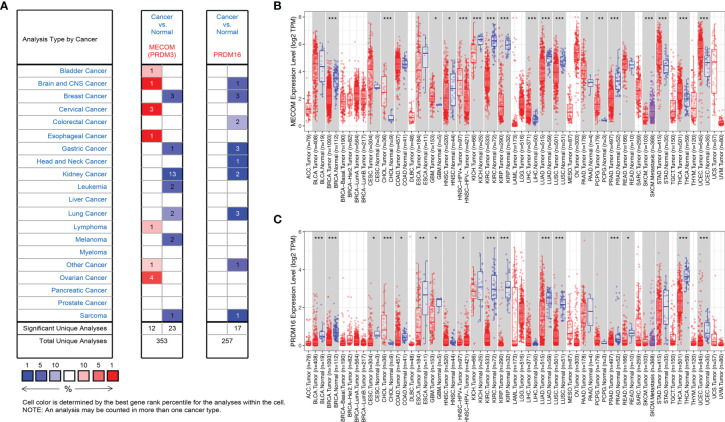
Expression of MECOM and PRDM16 in various tumors. **(A)** Expression of MECOM and PRDM16 in various tumors in the Oncomine database. Note: red and blue represent upregulation and downregulation of the target gene, respectively. **(B, C)** Expression of MECOM **(B)** and PRDM16 **(C)** in various tumors in the TIMER database. ^*^ *p* < 0.05; ^**^ *p* < 0.01; ^***^ *p* < 0.001. MECOM, MDS1 and EVI1 complex locus, also called PRDM3; PRDM16, PR domain containing 16, also called MEL1.

**Figure 2:**
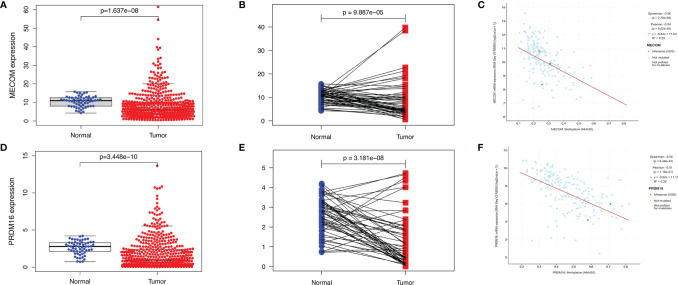
Expression of MECOM and PRDM16 in LUAD. **(A, D)** mRNA levels of MECOM **(A)** and PRDM16 **(D)** in LUAD tissues in TCGA. **(B, E)** MECOM **(B)** and PRDM16 **(E)** mRNA expression levels in paired tumor and adjacent normal tissues of 57 patients with LUAD in TCGA. **(C, F)** Correlation scatter plot of MECOM **(C)** and PRDM16 **(F)** mRNA expression and its methylation levels in LUAD in the cBioPortal database. MECOM, MDS1 and EVI1 complex locus, also called PRDM3; PRDM16, PR domain containing 16, also called MEL1; LUAD, lung adenocarcinoma.

As shown in **Figure 1A**, the results of Oncomine revealed that both MECOM and PRDM16 expression were downregulated in lung, breast, gastric, sarcoma, and kidney tumors compared with normal tissues (all *p* < 0.05). In addition, downregulation of MECOM and PRDM16 expression was also found in LUAD and lung squamous cell carcinomas (LUSC) in the TIMER database (**Figures 1B, C**, all *p* < 0.05). Further analysis of Oncomine elucidated that MECOM and PRDM16 were downexpressed in LUAD patients with a fold change of −2.081 and −3.069, respectively (**Table S3**). In addition, the expression of MECOM and PRDM16 in LUAD tissues were found to be significantly lower in the normal tissues by evaluating the 594 samples and 57 paired LUAD and adjacent noncancerous tissues in the TCGA database (**Figures 2A, B, D, E**, *p* < 0.05), and the low expression of the two genes were supposed to be related to the hypermethylation level of its DNA by CBioPortal analysis (**Figures 2C, F**).

### MECOM and PRDM16 Were Correlated With Clinicopathological Features and Prognosis in LUAD Patients

**Figure 3:**
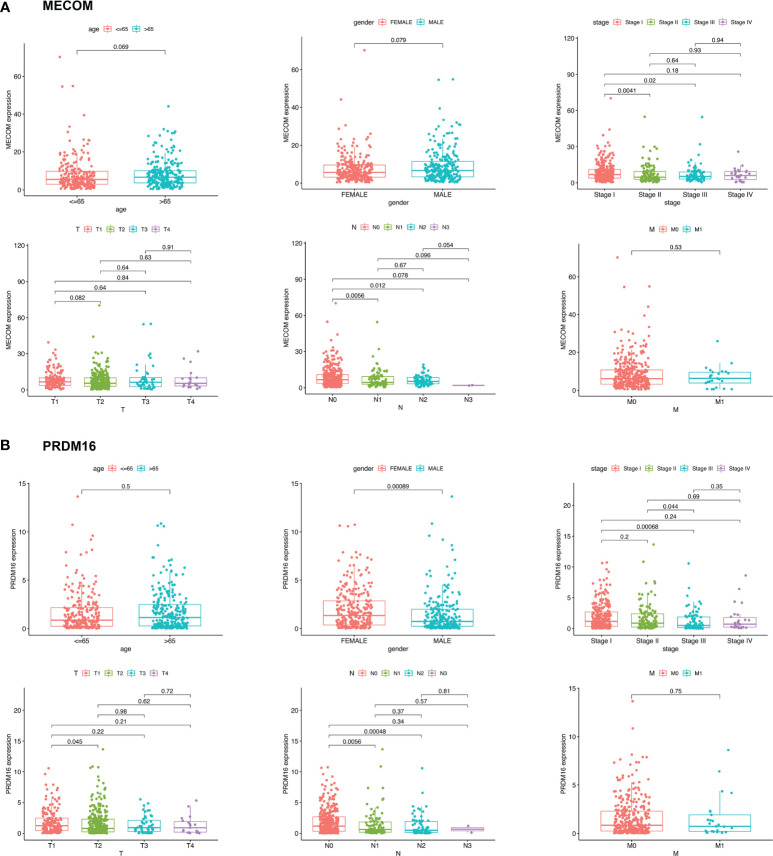
Expression levels of MECOM **(A)** and PRDM16 **(B)** in different clinicopathological characteristics of LUAD patients. MECOM, MDS1 and EVI1 complex locus, also called PRDM3; PRDM16, PR domain containing 16, also called MEL1; LUAD, lung adenocarcinoma.

**Figure 4:**
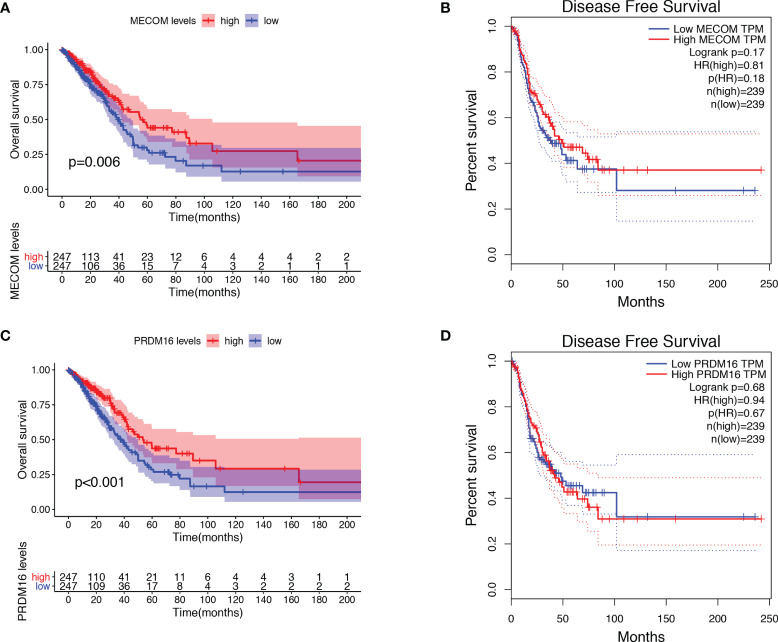
Correlation between the expression of MECOM and PRDM16 and prognosis in LUAD. **(A, B)** Correlation between MECOM, OS **(A)**, and DFS **(B)** in LUAD. **(C, D)** Correlation between PRDM16, OS **(C)**, and DFS **(D)** in LUAD. MECOM, MDS1 and EVI1 complex locus, also called PRDM3; PRDM16, PR domain containing 16, also called MEL1; LUAD, lung adenocarcinoma.

The results showed that low expression of MECOM and PRDM16 were correlated with poor pathological stage, high lymph node status, and poor OS in LUAD (**Figures 3A, B** and **4A, C**). Whereas, the expression of MECOM and PRDM16 had no effect on the DFS of LUAD (**Figures 4B, D**). In addition, MECOM and PRDM16 were also found to be associated with age, gender, race, stage, smoking status, tissue subtypes, TP53 mutation, and lymph node metastasis status in the UALCAN database (**Tables S4** and **S5**, all *p* < 0.05).

### Expression of MECOM and PRDM16 Correlated With Immune Cell Infiltration in LUAD

**Figure 5:**
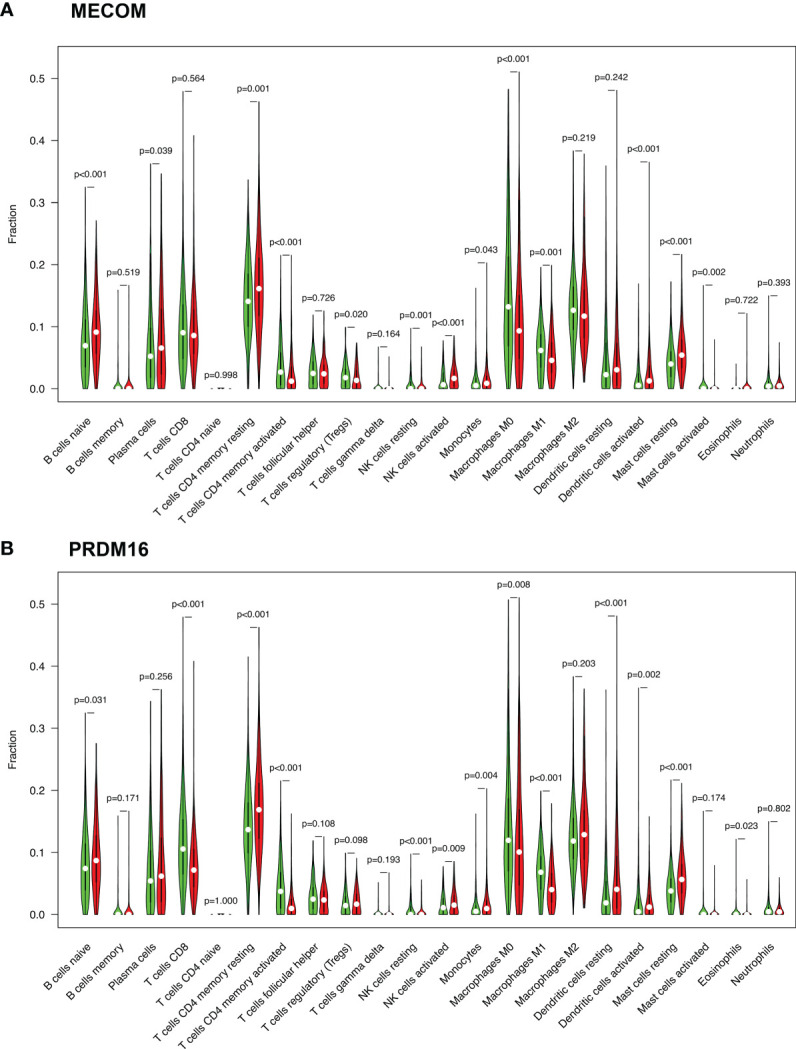
Expression of immune cells in LUAD patients with high and low expression of MECOM **(A)** and PRDM16 **(B)**. MECOM, MDS1 and EVI1 complex locus, also called PRDM3; PRDM16, PR domain containing 16, also called MEL1; LUAD, lung adenocarcinoma.

**Figure 6:**
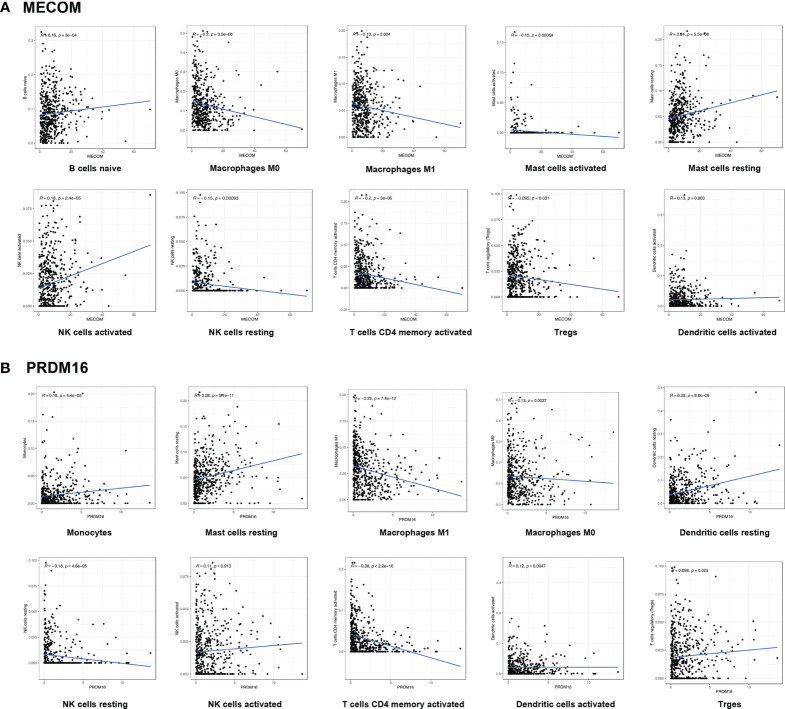
Correlation between MECOM **(A)**, PRDM16 **(B)** expression, and the level of immune cell infiltration in LUAD. MECOM, MDS1 and EVI1 complex locus, also called PRDM3; PRDM16, PR domain containing 16, also called MEL1; LUAD, lung adenocarcinoma.

We visualized the expression ratio of the immune cells in each LUAD sample from TCGA database and analyzed the correlation between different immune cells (**Figures S1A, B**). Some immune cells were differentially expressed between LUAD patients with high and low expression of MECOM and PRDM16 (**Figures 5A, B**). In addition, a significant positive relationship was found between MECOM and naive B cells, resting mast cells, activated NK cells, activated dendritic cells, plasma cells, and resting memory CD4 T cells (**Figure 6A**; **Figure S2A**; *p *<* *0.05). Macrophages M0 and M1, activated mast cells, resting NK cells, activated memory CD4 T cells, and Tregs were proved to be negatively correlated with MECOM (**Figure 6A**). As shown in **Figure 6B** and **Figure S2B**, there was a positive relationship between PRDM16 and monocytes, resting mast cells, resting and activated dendritic cells, activated NK cells, resting memory CD4 T cells, and Tregs. Whereas, macrophages M0 and M1, resting NK cells, activated memory CD4 T cells, eosinophils, and CD8 T cells were negatively correlated with PRDM16 (*p* < 0.05).

### MECOM- and PRDM16-Related Immune Cells Correlated With Prognosis in LUAD

**Figure 7:**
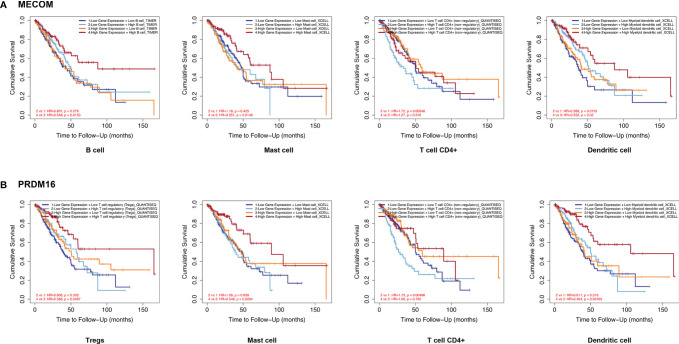
Correlation between MECOM- **(A)** and PRDM16-related **(B)** immune cell expression and prognosis in LUAD. MECOM, MDS1 and EVI1 complex locus, also called PRDM3; PRDM16, PR domain containing 16, also called MEL1; LUAD, lung adenocarcinoma.

Kaplan-Meier plots were constructed by the TIMER database to investigate the relationship between the expression of MECOM and PRDM16 and their related immune cells in the prognosis of LUAD. **Figure 7A** reveals that patients with high MECOM expression and high B-cell, mast cell, and myeloid dendritic cell levels were correlated with better prognosis (B cells: HR = 0.548, *p* = 0.015; mast cells: HR = 0.551, *p* = 0.015; myeloid dendritic cells: HR = 0.552, *p* = 0.020). However, patients with low levels of MECOM and high CD4^+^ T-cell levels seem to have a worse prognosis (HR = 1.720, *p* = 0.009). Among patient with high levels of PRDM16 and Tregs, mast cells and myeloid dendritic cells correlated with favorable outcomes (**Figure 7B**, Tregs: HR = 0.568, *p* = 0.047; mast cells: HR = 0.549, *p* = 0.026; myeloid dendritic cells: HR = 0.404, *p* = 0.001), and low expression of PRDM16 and high CD4^+^ T-cell levels was found to be associated with poor outcomes in LUAD (HR = 1.730, *p* =0.005).

### The Correlation Between MECOM, PRDM16, and Immune Cell Marker Expression

TIMER database was searched to assess the correlation between the expression of MECOM and PRDM16 and the levels of particular immune cell subset markers. As shown in **Table 1**, the results adjusted based on tumor purity revealed a significant correlation between MECOM and markers of monocyte (CD86), M1 macrophage (NOS2), M2 macrophage (COX2) neutrophils (CD66b, CCR7), NK (KIR2DL4), DC (HLA-DPB1, CD1C), Th1 (STAT1, IFNG), Th2 (STAT6, STAT5A), Tfh (BCL6), Th17 (STAT3), Treg (STAT5B), and T-cell exhaustion (PD-1, LAG3, HAVCR2, GZMB) in LUAD. PRDM16 was also demonstrated to be closely related to the markers of some immune cells including CD8^+^ T, monocyte, M2 macrophage, neutrophils, DC, NK, Th1, Th2, Tfh, Th17, Treg, and T-cell exhaustion in LUAD (**Table 1**).

**Table 1:** Correlation analysis between MECOM, PRDM16, and gene markers of immune cells in TIMER.

Description	Gene markers	MECOM				PRDM16			
		None		Purity		None		Purity	
		Cor	*p*-value	Cor	*p*-value	Cor	*p*-value	Cor	*p*-value
CD8^+^ T	CD8A	−0.044	3.14E−01	−0.043	3.49E−01	−0.168	1.30E−04^*^	−0.173	1.10E−04^*^
	CD8B	−0.080	7.08E−02	−0.066	1.43E−01	−0.166	1.58E−04^*^	−0.161	3.33E−04^*^
T cell (general)	CD3D	−0.077	8.06E−02	−0.064	1.53E−01	−0.075	8.89E−02	−0.084	6.18E−02
	CD3E	0.021	6.37E−01	0.057	2.09E−01	0.000	0.00	0.006	8.87E−01
	CD2	0.001	9.89E−01	−0.031	4.89E−01	−0.006	8.88E−01	−0.004	9.36E−01
B cell	CD19	0.011	8.02E−01	0.036	4.22E−01	0.076	8.32E−02	0.091	4.29E−01
	CD79A	−0.010	8.16E−01	0.012	7.86E−01	0.059	1.83E−01	0.076	9.16E−02
Monocyte	CD86	−0.122	5.43E−03^*^	−0.109	1.51E−02^*^	0.021	6.37E−01	0.029	5.18E−01
	CD115 (CSF1R)	−0.047	2.92E−01	−0.026	5.63E−01	0.153	4.75E−04^*^	0.168	1.78E−04^*^
TAM	CCL2	−0.087	4.83E−02	−0.083	6.50E−02	0.024	5.81E−01	0.016	7.16E−01
	CD68	−0.029	5.15E−01	−0.006	8.95E−01	−0.018	6.82E−02	−0.01	8.19E−01
	IL10	−0.055	2.15E−01	−0.039	3.83E−01	−0.029	5.05E−01	−0.04	3.74E−01
M1 macrophage	INOS (NOS2)	0.114	9.55E−03^*^	0.143	1.44E−03^*^	−0.023	6.04E−01	−0.017	7.01E−01
	IRF5	−0.090	4,18E−02	−0.076	9.05E−02	−0.023	6.09E−01	−0.019	6.67E−01
M2 macrophage	COX2 (PTGS2)	−0.083	6.08E−02	−0.089	4.75E−02^*^	0.140	1.44E−03^*^	0.147	1.05E−03^*^
	CD163	−0.008	8.48E−01	0.016	7.16E−01	0.017	6.94E−01	0.022	6.21E−01
	VSIG4	−0.019	3.85E−02	−0.071	1.15E−01	−0.002	9.71E−01	0.002	9.65E−01
	MS4A4A	−0.042	3.44E−01	−0.028	5.40E−01	0.021	6.27E−01	0.025	5.74E−01
Neutrophils	CD66b (CEACAM8)	0.174	7.12E−05^*^	0.187	3.01E−05^*^	0.260	2.14E−09^*^	0.261	4.30E−09^*^
	CD11b (ITGAM)	−0.026	5.50E−01	0.001	9.91E−01	0.138	1.68E−03^*^	0.150	8.06E−04^*^
	CCR7	0.076	8.44E−02	0.106	1.89E−02^*^	0.171	9.73E−05^*^	0.192	1.86E−05^*^
NK cell	KIR2DL1	0.064	1.45E−01	0.078	8.29E−02	−0.019	6.71E−01	−0.02	6.62E−01
	KIR2DL3	−0.020	6.48E−01	−0.005	9.10E−01	−0.088	4.55E−02	−0.087	5.38E−02
	KIR2DL4	−0.195	8.53E−06^*^	−0.175	9.14E−05^*^	−0.265	9.48E−10^*^	−0.267	1.84E−09^*^
	KIR3DL1	0.024	5.85E−01	0.045	3.24E−01	−0.062	1.62E−01	−0.075	9.79E−02
	KIR3DL2	−0.033	4.50E−01	−0.035	4.33E−01	−0.090	4.07E−02	−0.105	1.98E−02^*^
	KIR3DL3	−0.065	1.41E−01	−0.068	1.34E−01	−0.107	1.54E−02^*^	−0.110	1.45E−02^*^
	KIR2DS4	0.016	7.21E−01	0.034	4.53E−01	−0.039	3.78E−01	−0.052	2.51E−01
Dendritic cell	HLA-DPB1	0.068	1.23E−01	0.095	3.56E−02^*^	0.261	1.83E−09^*^	0.288	7.69E−11^*^
	HLA-DQB1	0.014	7.53E−01	0.036	4.23E−01	0.231	1.22E−07^*^	0.242	5.50E−08^*^
	HLA-DRA	0.002	9.70E−01	0.020	6.61E−01	0.178	4.65E−05^*^	0.197	1.00E−05^*^
	BDCA-1 (CD1C)	0.144	1.05E−03^*^	0.156	4.95E−04^*^	0.402	2.24E−21^*^	0.410	2.22E−21^*^
	BDCA-4 (NRP1)	−0.055	2.09E−01	−0.047	2.97E−01	0.119	6.76E−03^*^	0.115	1.04E−02^*^
	CD11c (ITGAX)	−0.032	4.66E−01	0.002	9.70E−01	0.083	6.09E−02	−0.102	2.29E−02^*^
	T-bet (TBX21)	0.013	7.65E−01	0.052	2.45E−01	−0.037	4.07E−01	−0.037	4.17E−01
Th1	STAT4	−0.025	5.73E−01	0.010	8.22E−01	0.034	4.46E−01	0.036	4.30E−01
	STAT1	−0.138	1.68E−03^*^	−0.111	1.35E−02^*^	−0.207	2.25E−06^*^	−0.209	2.91E−06^*^
	IFN-y (IFNG)	−0.161	2.52E−04^*^	−0.143	1.43E−03^*^	−0.265	1.07E−09^*^	−0.266	1.94E−09^*^
	TNF-a (TNF)	−0.082	6.41E−02	−0.080	7.63E−02	0.095	3.94E−02^*^	0.098	2.93E−02^*^
Th2	GATA3	−0.070	1.14E−01	−0.051	2.62E−01	−0.017	7.07E−01	−0.021	6.35E−01
	STAT6	0.392	2.07E−20^*^	0.401	1.98E−20^*^	0.307	9.90E−13^*^	−0.317	5.55E−13^*^
	STAT5A	0.074	9.48E−02	0.108	1.68E−02^*^	0.129	3.39E−03^*^	0.142	1.52E−03^*^
	IL13	0.027	5.46E−01	0.036	4.28E−01	0.039	3.82E−01	0.039	3.92E−01
Tfh	BCL6	0.240	3.55E−08^*^	0.248	2.57E−08^*^	0.201	4.4E−06^*^	0.205	4.33E−06^*^
	IL21	−0.06	1.71E−01	−0.042	3.48E−01	−0.116	8.4E−03^*^	−0.114	1.14E−02^*^
Th17	STAT3	0.379	5.13E−19^*^	0.397	4.24E−20^*^	0.336	4.86E−15^*^	0.333	2.94E−14^*^
	Il17A	0.024	5.83E−01	0.038	4.04E−01	−0.030	4.93E−01	−0.012	7.86E−01
Treg	FOXP3	−0.066	1.36E−01	−0.050	2.70E−01	0.025	5.68E−01	0.032	4.81E−01
	CCR8	0.024	5.38E−01	0.043	3.38E−01	0.053	2.27E−01	0.058	1.98E−01
	STAT5B	0.164	1.80E−04^*^	0.171	1.33E−04^*^	0.232	1.03E−07^*^	0.232	1.96E−07^*^
	TGFb (TGFb1)	−0.052	2.43E−01	−0.033	4.60E−01	0.169	1.11E−04^*^	0.178	7.41E−05^*^
T-cell exhaustion	PD-1 (PDCD1)	−0.160	2.74E−04^*^	−0.148	9.53E−04^*^	−0.101	2.23E−02^*^	−0.106	1.89E−02^*^
	CTLA4	−0.095	3.19E−02	−0.075	9.80E−02	−0.048	2.77E−01	−0.052	2.49E−01
	LAG3	−0.141	1.38E−03^*^	−0.120	7.41E−03^*^	−0.114	9.94E−03^*^	−0.107	1.70E−02^*^
	TIM-3 (HAVCR2)	−0.121	5.83E−03^*^	−0.110	1.48E−02^*^	−0.029	5.07E−01	−0.028	5.39E−01
	GZMB	−0.225	2.34E−07^*^	−0.209	2.71E−06^*^	−0.337	3.68E−15^*^	−0.358	2.56E−16^*^

Cor, R-value of Spearman’s correlation. None, correlation without adjustment. Purity, correlation adjusted by purity. ^*^p < 0.05.

LUAD, lung adenocarcinoma; TAM, tumor-correlated macrophage; Tfh, follicular helper T cell; Th, T helper cell; Treg, regulatory T cell.

### Identification of MECOM and PRDM16 Coexpressed Genes

**Figure 8:**
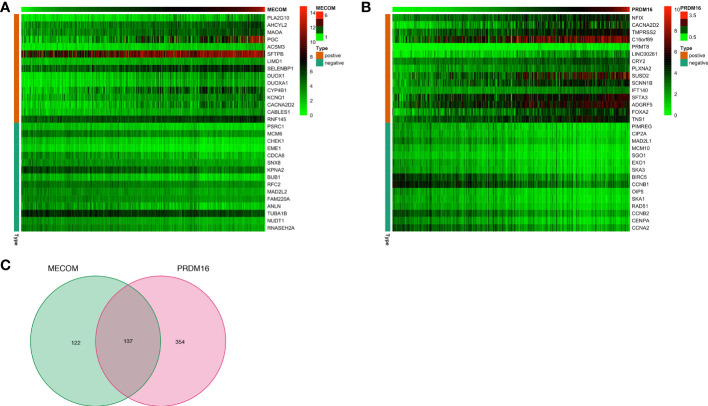
The top 15 coexpression genes of MECOM **(A)** and PRDM16 **(B)** in LUAD in the cBioPortal database. **(C)** Intersection coexpression genes of MECOM and PRDM16. Note: Genes with |r| > 0.4 and *p* < 0.001 were considered coexpression genes. MECOM, MDS1 and EVI1 complex locus, also called PRDM3; PRDM16, PR domain containing 16, also called MEL1; LUAD, lung adenocarcinoma.

In the CBioPortal database, 103 and 156 genes were positively and negatively correlated with MECOM, respectively. In addition, 215 positively and 276 negatively correlated genes with PRDM16 were also identified. The heatmap (**Figures 8A, B**) showed the top 15 positively and negatively correlated genes with MECOM and PRDM16. A total of 137 intersections of the two coexpressed genes were further explored by the Venn diagram (**Figure 8C**).

### The Pathways Regulated by MECOM and PRDM16 in LUAD Were Revealed by GO and KEGG Analyses

**Figure 9:**
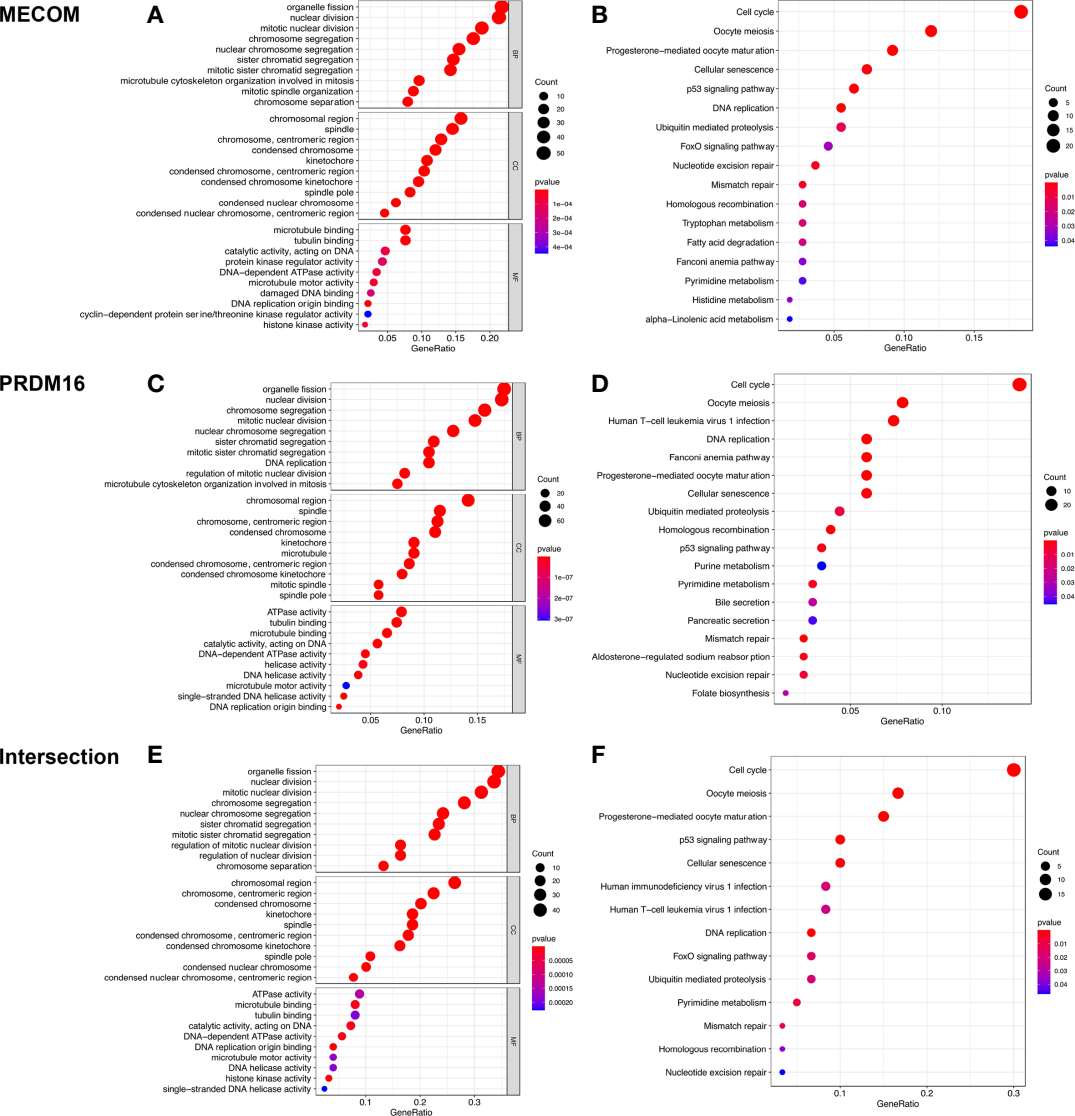
GO and KEGG analyses of MECOM **(A, B)** and PRDM16 **(C, D)** coexpression genes and intersection of coexpression genes **(E, F)**. MECOM, MDS1 and EVI1 complex locus, also called PRDM3; PRDM16, PR domain containing 16, also called MEL1; LUAD, lung adenocarcinoma.

GO analysis elucidated that MECOM- and PRDM16-coexpressed genes were mainly involved in mitosis, DNA replication, and organelle division (**Figures 9A, C, E**). Coexpressed genes were also found enriched in regulating cell cycle, cellular senescence, DNA replication, and p53 signaling pathway by KEGG analysis (**Figures 9B, D, F**).

### MECOM- and PRDM16-Related Hub Genes in PPI Network

**Figure 10:**
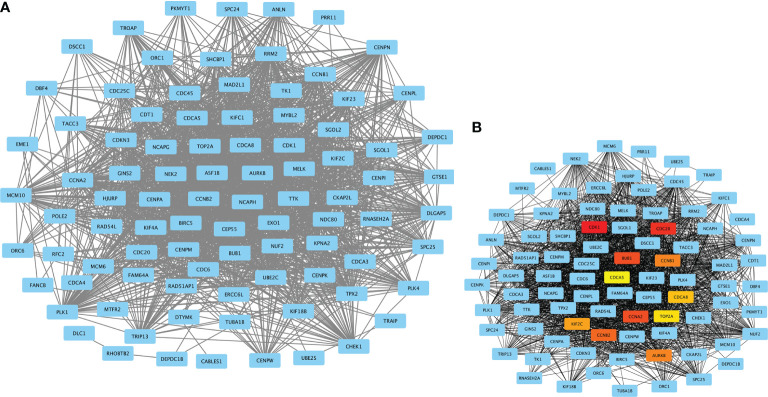
PPI network constructed by MECOM and PRDM16 intersection coexpression genes. **(A)** PPI network. **(B)** Ten hub genes in the PPI network. The top 10 hub genes are shown in different colors except blue. MECOM, MDS1 and EVI1 complex locus, also called PRDM3; PRDM16, PR domain containing 16, also called MEL1; LUAD, lung adenocarcinoma.

**Figure 11:**
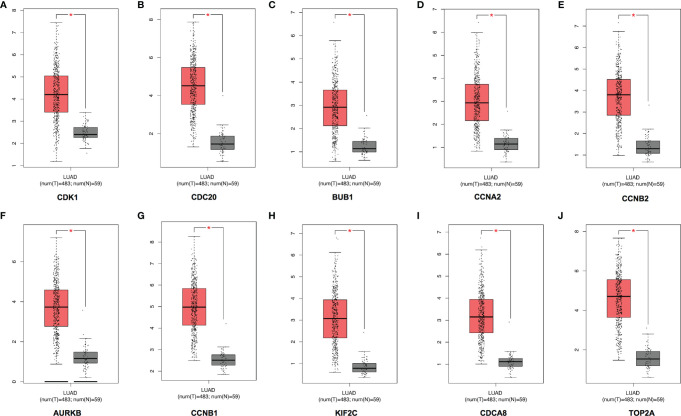
Expression of the top 10 hub genes in LUAD in the GEPIA database. The expressions of CDK1 **(A)**, CDC20 **(B)**, BUB1 **(C)**, CCNA2 **(D)**, CCNB2 **(E)**, AURKB **(F)**, CCNB1 **(G**), KIF2C **(H)**, CDCA8 **(I)**, and TOP2A **(J)** in LUAD. LUAD, lung adenocarcinoma. *p < 0.05.

**Figure 12:**
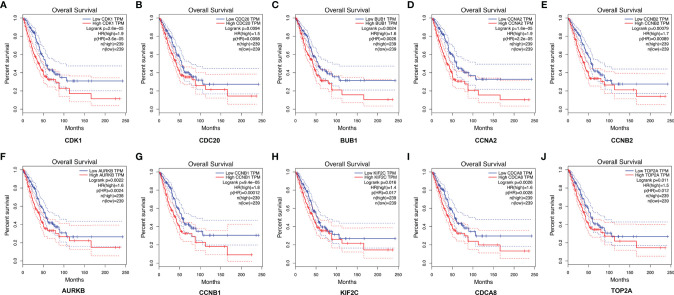
The correlation between the top 10 hub genes and the OS of LUAD patients in the GEPIA database. CDK1 **(A)**, CDC20 **(B)**, BUB1 **(C)**, CCNA2 **(D)**, CCNB2 **(E)**, AURKB **(F**), CCNB1 **(G)**, KIF2C **(H)**, CDCA8 **(I)**, and TOP2A **(J)**. LUAD, lung adenocarcinoma.

A total of 10 hub genes (CDK1, CDC20, BUB1, CCNA2, CCNB2, AURKB, CCNB1, KIF2C, CDCA8, TOP2A) identified by PPI network analysis (**Figures 10A, B**; **Table 2**) were observed to be elevated in LUAD and related to poor OS by the GEPIA database (**Figures 11A–J** and **12A–J**). Additionally, CDK1, AURKB, BUB1, CCNB1, and CCNB2 were also demonstrated to correlate with the DFS of LUAD patients (**Figure S3**).

**Table 2:** The top 10 hub genes in the protein-protein interaction (PPI) network ranked by degree method.

Gene symbol	Gene description	Score
CDK1	Cyclin-dependent kinase 1	75
CDC20	Cell division cycle 20	74
BUB1	Budding uninhibited by benzimidazoles 1 homolog	73
CCNA2	Cyclin A2	73
CCNB2	Cyclin B2	72
AURKB	Aurora kinase B	71
CCNB1	Cyclin B1	71
KIF2C	Kinesin family member 2C	70
CDCA8	Cell division cycle associated 8	69
TOP2A	Topoisomerase (DNA) II alpha 170 kDa	67

### Verification of MECOM and PRDM16 Expression in LUAD Tissue Samples

**Figure 13:**
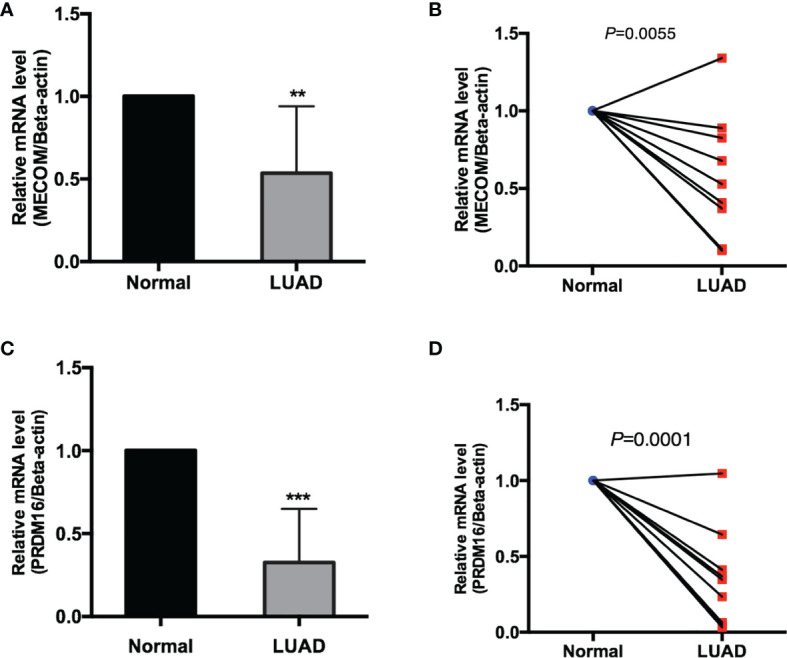
The mRNA expression of MECOM **(A, B)** and PRDM16 **(C, D)** in clinical samples of LUAD. MECOM, MDS1 and EVI1 complex locus, also called PRDM3; PRDM16, PR domain containing 16, also called MEL1; LUAD, lung adenocarcinoma. **p < 0.01; ***p < 0.001.

The mRNA expression of MECOM and PRDM16 was evaluated in LUAD patients’ tissue by qRT-PCR. As shown in **Figures 13A–D**, both MECOM and PRDM16 were found to be downregulated in primary tumorous tissues of LUAD patients compared with the adjacent noncancerous tissues (*p* < 0.05), which is consistent with the results of the bioinformatic analysis.

## Discussion

The PRDM family was illustrated to participate in a series of important cellular processes, and the abnormal function of some members may cause malignant transformation. In fact, we systematically investigate the expression of all members of the PRDM family and the relationship between them and prognosis in LUAD at the early stage of the study based on the above background. We found that only two members, MECOM and PRDM16, were differentially expressed and correlated with the prognosis in LUAD. Many kinds of literature further confirm that MECOM and PRDM16 are two highly correlated transcription factors and play an accumulative role in some biological functions and the pathogenesis of the disease (10, 11). MECOM encodes PRDM3, which is involved in the process of hematopoiesis, apoptosis, development, differentiation, and proliferation (26–29) and considered an oncoprotein in the hematopoietic system and has been found to promote leukemogenesis by overexpression of the AML1 gene through the translocation of AML1 (30). Whereas, MECOM has been found to function as both oncogene and tumor suppressor gene (TSG) in some solid tumors (13), which was observed to be overexpressed in colon and breast cancer specimens and correlated with worse outcomes (31, 32), by contrast, the favorable prognosis of ovarian cancer patients were identified to be associated with amplification of MECOM gene (33). PRDM16 is highly homologous to MECOM, and they have been found to be together involved in the maintenance of hematopoietic stem cell function and heterochromatin integrity (10, 34). Additionally, amplification of PRDM16 was found in leukemia (35), osteosarcoma (36), and gastric cancer (37), and reduced expression of PRM16 was observed in uterine leiomyosarcoma (38) and NSCLC (18, 39), which elucidated PRDM16 and also functions as an oncogene or TSG in several cancers as MECOM. Together, these data suggest that alterations of MECOM and PRDM16 are possibly involved in tumorigenesis by either upregulation or downregulation. Although research demonstrated that PRDM16 can inhibit metastasis of LUAD (18), the relationship between PRDM16, prognosis, and immune infiltration in LUAD were not further evaluated. In addition, the role of MECOM in LUAD has not been studied.

In the present study, we found that MECOM and PRDM16 were downregulated and related to age, gender, race, smoking status, pathological stage, N stage, TP53 mutation status, and poor OS in LUAD. Previous studies also found that MECOM and PRDM16 were identified to be involved in the development and drug resistance in leukemia by coupling with p53 (40, 41). In addition, the coexpressed genes of the MECOM and PRDM16 were further found to be enriched in regulating cell cycle, cellular senescence, DNA replication, and p53 signaling pathway; these are the classic pathways involved in tumorigenesis.

The tumor immune microenvironment (TIME), including the composition, distribution, and expression of various biomarkers of tumor immune cells, plays a crucial role in the progression and treatment response of LUAD (42). Hence, immunotherapy has become a new direction of tumor therapy. Herein, the correlation between the MECOM and PRDM16 expression and the infiltration of the immune cells were further investigated to evaluate whether the two genes influence the prognosis by participating in the immune response to LUAD. We found that MECOM and PRDM16 were closely associated with multiple immune cell infiltration such as macrophages, mast cells, T cells, B cells, and NK cells. The expression of MECOM, PRDM16, and immune cells together affect the prognosis of patients with LUAD. Similar to our findings, PRDM3 decreases pancreatic tumorigenesis by regulating immune cell activation and infiltration (16). Whereas, the function of PRDM16 in the immune response of cancers was not systematically investigated, which needs to be further studied.

We also identified 10 hub genes (CDK1, CDC20, BUB1, CCNA2, CCNB2, AURKB, CCNB1, KIF2C, CDCA8, TOP2A) that were overexpressed in LUAD and associated with poor survival, among which, CDK1, AURKB, BUB1, CCNB2, and CCNB were also correlated with the DFS of LUAD patients. Notably, we further validated the expression of MECOM and PRDM16 in clinical specimens, which is consistent with the results of the bioinformatics analysis.

The advantage of this study is that the expression of the two highly related PRDM members, MECOM and PRDM16, and the correlation between them, prognosis, and immune cell infiltration in the LUAD were explored for the first time. Importantly, the results were further verified in the clinical samples. Whereas, there are still some limitations: (i) the sample size of the clinical sample is insufficient and (ii) the molecular biology experiment was not conducted to investigate the function of the two genes in LUAD. Considering the importance of the two genes in the prognosis and immune cell infiltration of LUAD, it is worthwhile to further study the mechanism of the two genes in regulating the occurrence and development of LUAD in the future.

## Conclusion

These findings together support the notion that MECOM and PRDM16 are potential immune-related and prognostic biomarkers for LUAD, and the mechanism of MECOM and PRDM16 in LUAD deserves in-depth investigation by molecular biology experiment.

## Data Availability Statement

The raw data supporting the conclusions of this article will be made available by the authors, without undue reservation.

## Ethics Statement

The studies involving human participants were reviewed and approved by the Ethics Committee of the First Affiliated Hospital of Xi’an Jiaotong University. The patients/participants provided their written informed consent to participate in this study.

## Author Contributions

Conception and design: PS and MC. Administrative support: PS and MC. Provision of study materials or patients: ML, HR, YZ, NL, MF, KW, and TY. Experiment operations, collection, and assembly of data: ML, HR, YZ, NL, MF, KW, and TY. Data analysis and interpretation: ML, HR, YZ, NL, MF, KW, TY, MC, and PS. Manuscript writing: all authors. Final approval of manuscript: all authors. All authors listed have made a substantial, direct, and intellectual contribution to the work and approved it for publication.

## Funding

The research was funded by the following projects: the Key research and development projects of Shaanxi Province (No. 2018KW-039; No. 2021ZDLSF02-05; No. 2019-KW-034); the National Natural Science Foundation of China (No. 81802291; No. 82103467); Fundamental Research Funds for the Central Universities in Xi’an Jiaotong University (No. 1191320149; No. xzy012021064); and China Postdoctoral Science Foundation Grant (No. 2019M653666).

## Conflict of Interest

The authors declare that the research was conducted in the absence of any commercial or financial relationships that could be construed as a potential conflict of interest.

## Publisher’s Note

All claims expressed in this article are solely those of the authors and do not necessarily represent those of their affiliated organizations, or those of the publisher, the editors and the reviewers. Any product that may be evaluated in this article, or claim that may be made by its manufacturer, is not guaranteed or endorsed by the publisher.

## Supplementary Material

Supplementary Figure S1 Proportion of immune cells **(A)** and correlation between immune cells **(B)** in LUAD samples in TCGA database. LUAD, Lung adenocarcinoma.

Supplementary Figure S2 Correlation between MECOM **(A)**, PRDM16 **(B)** expression and the level of immune cells infiltration in LUAD. MECOM, MDS1 and EVI1 complex locus, also called PRDM3; PRDM16, PR domain containing 16, also called MEL1; LUAD, Lung adenocarcinoma.

Supplementary Figure S3 The correlations of the top 10 Hub genes with DFS of LUAD patients in the GEPIA database. CDK1 **(A)**, CDC20 **(B**), BUB1 **(C)**, CCNA2 **(D)**, CCNB2 **(E)**, AURKB **(F)**, CCNB1 **(G)**, KIF2C **(H)**, CDCA8 **(I)** and TOP2A **(J)**. DFS, Disease-free survival.
