# Supplementary figures and images for: MECOM/PRDM3 and PRDM16 Serve as Prognostic-Related Biomarkers and Are Correlated With Immune Cell Infiltration in Lung Adenocarcinoma
Source: Front Oncol. 2022 Jan 31;12:772686. doi: 10.3389/fonc.2022.772686 (PMC8841357; doi:10.3389/fonc.2022.772686)

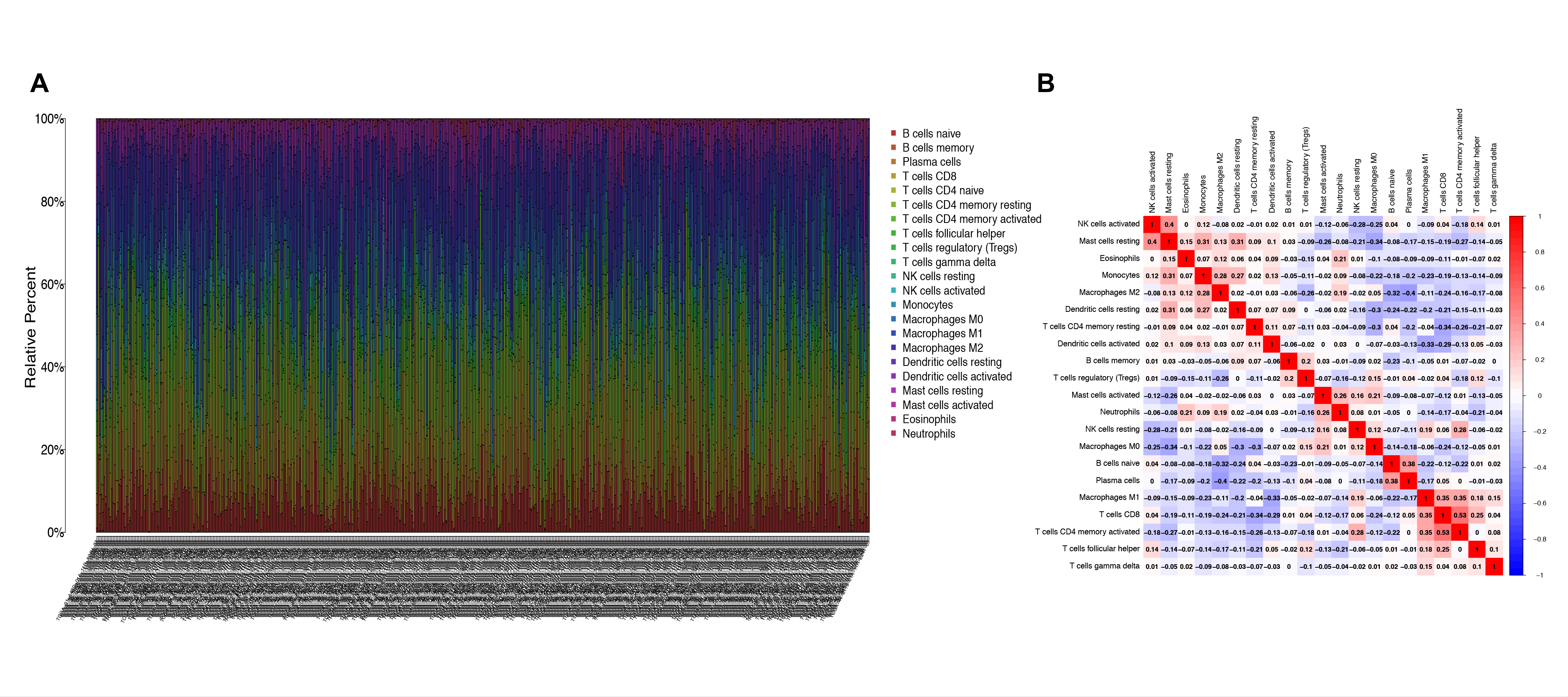

Supplement: Supplementary Figure S1 — Proportion of immune cells (A) and correlation between immune cells (B) in LUAD samples in TCGA database. LUAD, Lung adenocarcinoma. [file Image_1.tif]

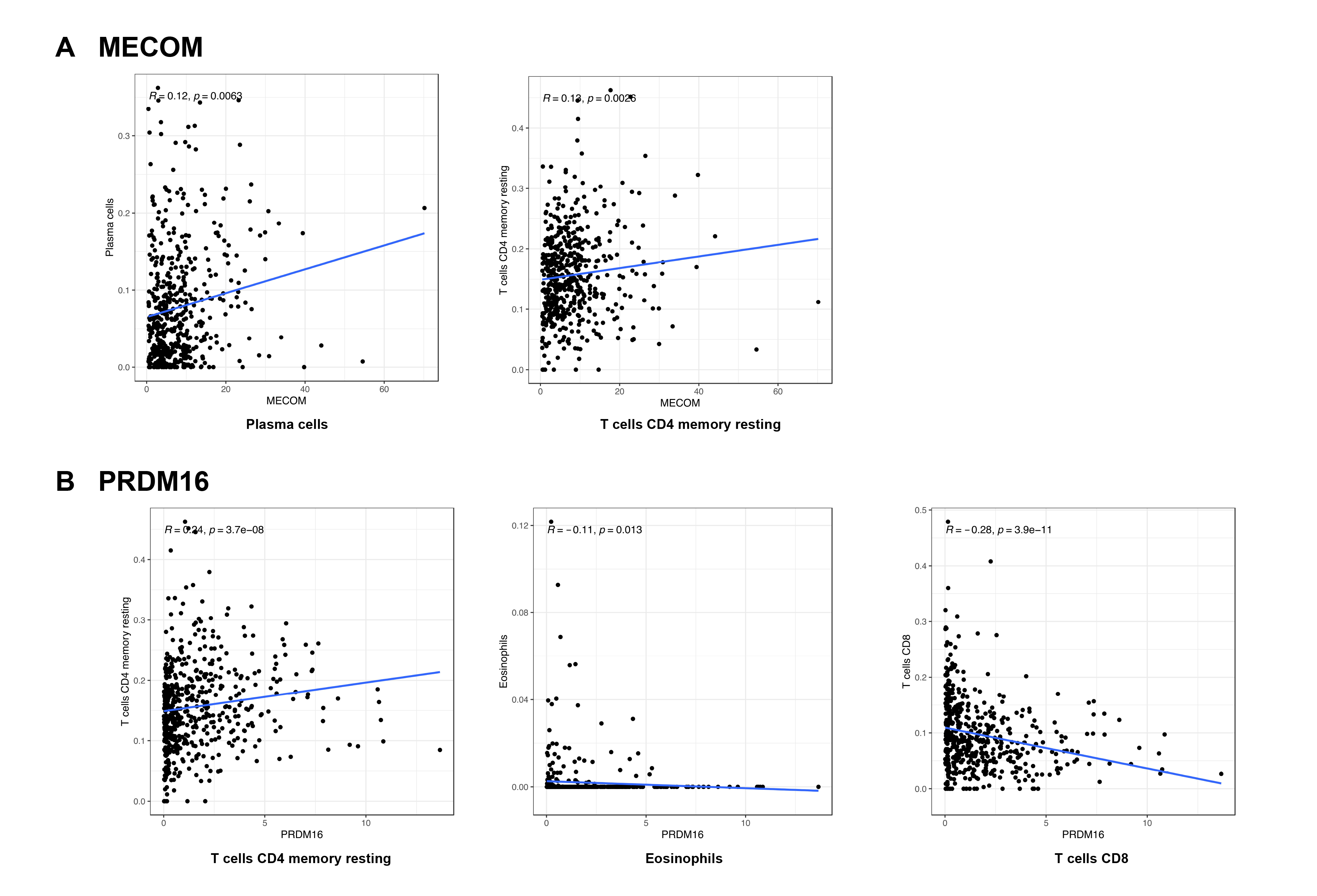

Supplement: Supplementary Figure S2 — Correlation between MECOM (A), PRDM16 (B) expression and the level of immune cells infiltration in LUAD. MECOM, MDS1 and EVI1 complex locus, also called PRDM3; PRDM16, PR domain containing 16, also called MEL1; LUAD, Lung adenocarcinoma. [file Image_2.tif]

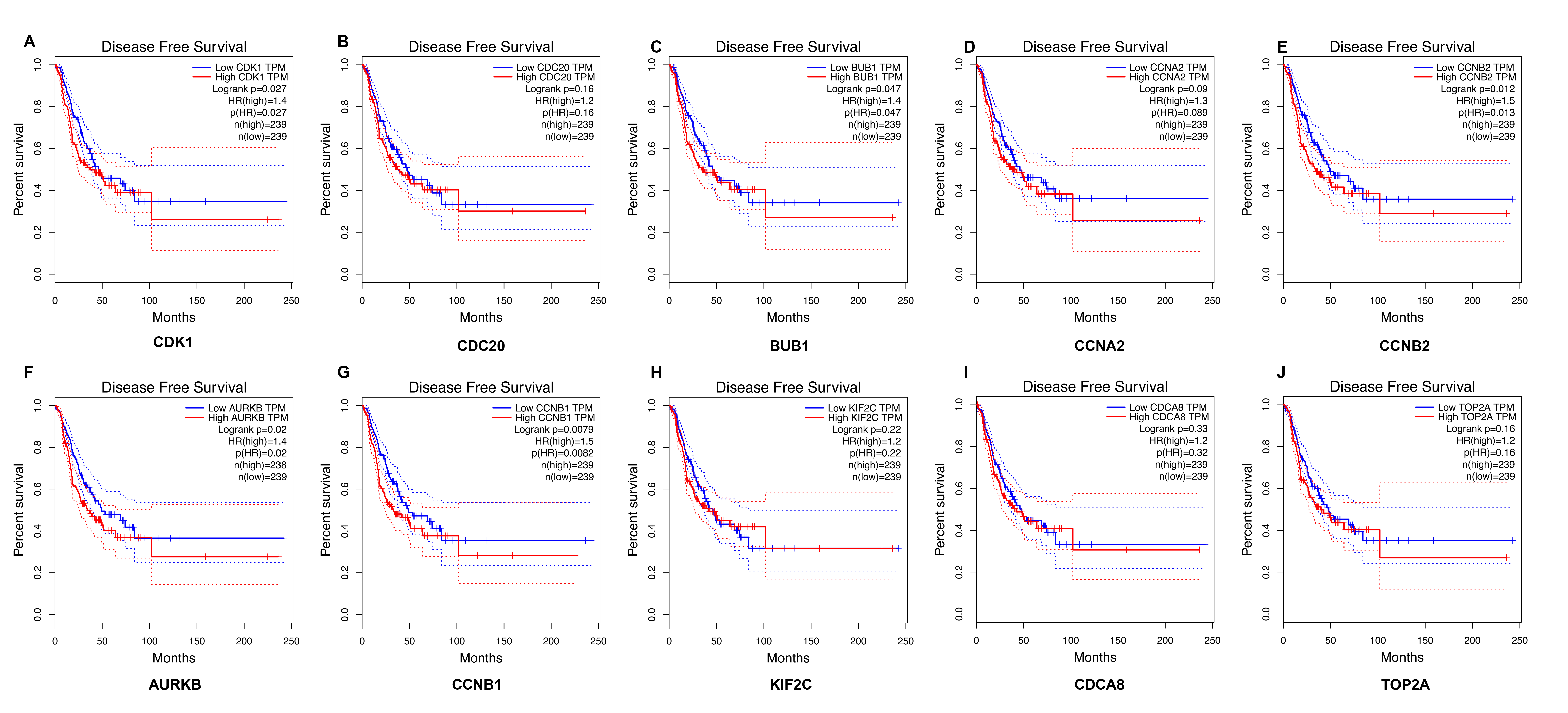

Supplement: Supplementary Figure S3 — The correlations of the top 10 Hub genes with DFS of LUAD patients in the GEPIA database. CDK1 (A), CDC20 (B), BUB1 (C), CCNA2 (D), CCNB2 (E), AURKB (F), CCNB1 (G), KIF2C (H), CDCA8 (I) and TOP2A (J). DFS, Disease-free survival. [file Image_3.tif]
