# Supplementary material for: MECOM/PRDM3 and PRDM16 Serve as Prognostic-Related Biomarkers and Are Correlated With Immune Cell Infiltration in Lung Adenocarcinoma
Source: Front Oncol. 2022 Jan 31;12:772686. doi: 10.3389/fonc.2022.772686 (PMC8841357; doi:10.3389/fonc.2022.772686)
Supplement: Supplementary file 4 [file Table_1.docx]

**Table S1**  Clinicopathological characteristics of the 522 LUAD patients from TCGA database.

| **Clinical Characteristics** | **Total (N=522)** | **Percentage(%)** |
| --- | --- | --- |
| Age at diagnosis | 66 (59-73) |  |
| **Gender** |  |  |
| Female | 280 | 53.6 |
| Male | 242 | 46.4 |
| **Clinical stage** |  |  |
| I | 279 | 54.3 |
| II | 124 | 24.1 |
| III | 85 | 16.5 |
| IV | 26 | 5.1 |
| **T stage** |  |  |
| 1 | 172 | 33.1 |
| 2 | 281 | 54.1 |
| 3 | 47 | 9.1 |
| 4 | 19 | 3.7 |
| **Lymph nodes** |  |  |
| 0 | 335 | 65.7 |
| 1 | 98 | 19.2 |
| 2 | 75 | 14.7 |
| 3 | 2 | 0.4 |
| **Distant metastasis** |  |  |
| Negative | 353 | 93.4 |
| Positive | 25 | 6.6 |

LUAD, Lung adenocarcinoma.

**Table S2** Information of primer sequences of qRT-PCR.

| Gene | Forward primer | Reverse primer |
| --- | --- | --- |
| MECOM | 5'-AAACTCGAAAGCGAGAATGATCT-3' | 5'-TGGTGGCGAATTAAATTGGACTT-3' |
| PRDM16 | 5'-CGAGGCCCCTGTCTACATTC-3' | 5'- GCTCCCATCCGAAGTCTGTC-3' |
| Beta-actin | 5'-GAAGAGCTACGAGCTGCCTGA-3' | 5'-CAGACAGCACTGTGTTGGCG-3' |

MECOM,MDS1 and EVI1 complex locus, also known as PRDM3; PRDM16, PR domain containing 16, also known as MEL1;LUAD, lung adenocarcinoma.

**Table S3** The expression of MECOM and PRDM16 in LUAD in Oncomine database

| Gene | Type of LUAD vs. Normal | Fold change | t-test | *P*-value | Reference |
| --- | --- | --- | --- | --- | --- |
| MECOM | Lung Adenocarcinoma vs. Normal | -2.081 | -8.545 | 7.57E-13 | Landi |
| PRDM16 | Lung Adenocarcinoma vs. Normal | -3.069 | -7.864 | 4.00E-11 | Hou |

MECOM,MDS1 and EVI1 complex locus, also known as PRDM3; PRDM16, PR domain containing 16; LUAD, lung adenocarcinoma.

**Table S4** The correlation between MECOM and clinicopathologic features in LUAD patients in the UALCAN database

| Clinicopathologic features | N | MECOM (transcript per million)  Median (interquartile range) | *P*-value |
| --- | --- | --- | --- |
| Age (years) |  |  |  |
| 21-40 vs. 41-60 | 12 vs. 90 | 1.804(1.762,1.969) vs. 13.752(7.814,21.704) | 1.41E-04^***^ |
| 21-40 vs. 61-80 | 12 vs. 149 | 1.804(1.762,1.969) vs. 18.039(9.038-29.014) | 3.77E-04^***^ |
| 21-40 vs. 81-100 | 12 vs. 32 | 1.804(1.762,1.969) vs. 20.407(12.881,26.158) | 4.37E-05^***^ |
| Gender |  |  |  |
| Male vs. Female | 238 vs. 276 | 18.701 (8.839,29.816) vs. 16.024 (8.839,15.974) | 1.94E-02^*^ |
| Race |  |  |  |
| Caucasian vs. African American | 387 vs. 51 | 17.261 (8.811,27.694) vs. 14.257 (6.865,25.546) | 4.40E-04^***^ |
| Smoking |  |  |  |
| Non smoker vs. Reformed Smoker1 | 75 vs. 135 | 16.149(9.899,24.148) vs. 21.954(12.434,68.192) | 3.62E-02^*^ |
| Smoker vs. Reformed Smoker1 | 118 vs. 135 | 10.432(5.976,19.218) vs. 21.954(12.434,68.192) | 3.41E-04^***^ |
| Reformed Smoker1 vs. Reformed Smoker2 | 135 vs. 168 | 21.954(12.434,68.192) vs. 18.877(9.494,27.085) | 1.63E-02^*^ |
| Stage |  |  |  |
| Stage 1 vs. Stage 2 | 277 vs. 125 | 18.848 (10.05,29.692) vs. 12.537 (7.521,23.941) | 6.31 E-02 |
| Stage 1 vs. Stage 3 | 277 vs. 85 | 18.848 (10.05,29.692) vs. 14.825 (8.831,24.994) | 9.11 E-01 |
| Stage 1 vs. Stage 4 | 277 vs. 28 | 18.848 (10.05,29.692) vs. 15.780 (8.568,26.449) | 8.32 E-01 |
| Stage 2 vs. Stage 3 | 125 vs. 85 | 12.537 (7.521,23.941) vs. 14.825 (8.831,24.994) | 9.21 E-01 |
| Stage 2 vs. Stage 4 | 125 vs. 28 | 12.537 (7.521,23.941) vs. 15.780 (8.568,26.449) | 7.15 E-01 |
| Stage 3 vs. Stage 4 | 85 vs. 28 | 14.825 (8.831,24.994) vs. 15.780 (8.568,26.449) | 7.84 E-01 |
| Tissue subtype |  |  |  |
| NOS vs. Mixed | 320 vs. 107 | 13.726 (7.126,24.879) vs. 17.860(10.304,30.600) | 8.45 E-03^**^ |
| NOS vs. LBC NonMucinous | 320 vs. 19 | 13.726 (7.126,24.879) vs. 24.144 (19.116,32.641) | 3.16 E-02^*^ |
| NOS vs. Solid pattern Predominant | 320 vs. 5 | 13.726 (7.126,24.879) vs. 8.279 (2.169,8.627) | 7.23 E-04^***^ |
| NOS vs. LBC Mucinous | 320 vs. 5 | 13.726 (7.126,24.879) vs. 40.234 (39.324,41.265) | 2.58 E-02^*^ |
| NOS vs. Papillary | 320 vs. 23 | 13.726 (7.126,24.879) vs. 26.723 (18.918,47.303) | 1.19 E-02^*^ |
| Mixed vs. Solid pattern Predominant | 107 vs. 5 | 17.860 (10.304,30.600) vs. 8.279 (2.169,8.627) | 1.77E-07^***^ |
| Mixed vs. Mucinous | 107 vs. 5 | 17.860 (10.304,30.600) vs. 93.848(74.216,113.480 ) | 7.62E-04^***^ |
| Clear Cell vs. Solid Pattern Predominant | 2 vs. 5 | 17.744(15.14,20.347) vs. 8.279 (2.169,8.627) | 4.28E-02^*^ |
| LBC NonMucinous vs. Solid Pattern Predominant | 19 vs. 5 | 24.144(19.116,332.641) vs. 8.279 (2.169,8.627) | 6.41E-05^***^ |
| Solid Pattern Predominant vs. Acinar | 5 vs. 18 | 8.279 (2.169,8.627) vs. 18.937(14.375,29.852) | 7.41E-04^***^ |
| Solid Pattern Predominant vs. LBC NonMucinous | 5 vs.19 | 8.279 (2.169,8.627) vs. 24.144 (19.116,32.641) | 1.48E-02^*^ |
| Solid Pattern Predominant vs. Mucinous carcinoma | 5 vs. 10 | 8.279 (2.169,8.627) vs. 27.260 (20.142,39.38) | 1.80E-03^**^ |
| Solid Pattern Predominant vs. Papillary | 5 vs. 23 | 8.279 (2.169,8.627) vs. 26.723 (18.918,47.303) | 6.00E-05^***^ |
| Papillary vs. Mucinous | 23 vs. 2 | 26.723 (18.918,47.303) vs. 93.848(74.216,113.480 ) | 2.07E-02^*^ |
| Nodal metastasis status |  |  |  |
| N0 vs. N1 | 331 vs. 96 | 18.701 (9.507,29.284) vs. 12.115 (7.722,23.395) | 4.95E-02^*^ |
| N0 vs. N2 | 96 vs. 74 | 18.701 (9.507,29.284) vs. 14.84 (8.896,24.014) | 2.26E-05^***^ |
| TP53 mutation status |  |  |  |
| Mutation vs. Non-mutation | 223 vs. 279 | 13.225 (7.047,21.028 vs. 20.674 (10.561,31.924) | 8.72E-08^***^ |

MECOM, MDS1 and EVI1 complex locus, Alias: PRDM3; PRDM16, PR domain containing 16; LUAD, lung adenocarcinoma; Reformed Smoker 1, <15 years；Reformed Smoker 2, >15 years; NOS, lung adenocarcinoma-not otherwise specified; LBC NonMucinous, Lung Bronchioloavleolar Noncarcinmoma; LBC Mucinous, Lung Bronchioloavleolar Carcinoma Mucinous; Papillary, lung papillary adenocarcinoma; Mucinous, mucinous (colloid) carcinoma; Clear cell, Lung clear cell Adenocarcinoma; Mucinous, lung mucinous adenocarcinoma; Mixed, lung adenocarcinoma mixed subtype. ^***^*P*<0.001, ^**^*P*<0.01, ^*^*P*<0.05.

**Table S5** The correlation between PRDM16 and clinicopathologic features in LUAD patients in the UALCAN database

| Clinicopathologic features | N | PRDM16 (transcript per million)  Median (interquartile range) | *P*-value |
| --- | --- | --- | --- |
| Age (years) |  |  |  |
| 21-40 vs. 41-60 | 12 vs. 90 | 0.28(0.177,0.457) vs. 1.155(0.331,3.332) | 2.26E-10^***^ |
| 21-40 vs. 61-80 | 12 vs. 149 | 0.28(0.177,0.457)vs. 1.468(0.385-3.395) | 8.24E-12^***^ |
| 21-40 vs. 81-100 | 12 vs. 32 | 0.28(0.177,0.457) vs. 0.837(0.342,3.489) | 8.78E-04^***^ |
| Gender |  |  |  |
| Male vs. Female | 238 vs. 276 | 0.92 (0.278,2.387) vs. 1.904 (0.467,4.093) | 5.34E-03^**^ |
| Race |  |  |  |
| Caucasian vs. African American | 387 vs. 51 | 1.457 (0.404,3.666) vs. 1.036 (0.21,2.588) | 5.73E-01 |
| Smoking |  |  |  |
| Non smoker vs. Smoker | 75 vs. 135 | 2.87(0.847,4.566) vs. 0.953(0.249,2.651) | 8.40E-03^**^ |
| Non smoker vs.Reformed Smoker2 | 75 vs. 168 | 2.87(0.847,4.566) vs. 0.911(0.239, 2.587) | 1.32E-03^*^ |
| Smoker vs. Reformed Smoker1 | 118 vs. 135 | 0.953(0.249,2.651) vs. 1.995(0.593,4.748) | 5.64E-03^**^ |
| Reformed Smoker1 vs. Reformed Smoker2 | 135 vs. 168 | 1.995(0.593,4.748) vs. 0.911(0.239, 2.587) | 1.83E-04^***^ |
| Stage |  |  |  |
| Stage 1 vs. Stage 2 | 277 vs. 125 | 1.597 (0.448,3.726) vs. 1.288(0.325,3.437) | 4.53E-1 |
| Stage 1 vs. Stage 3 | 277 vs. 85 | 1.597 (0.448,3.726) vs. 0.713 (0.218,2.781) | 4.52 E-03^**^ |
| Stage 1 vs. Stage 4 | 277 vs. 28 | 1.597 (0.448,3.726) vs. 1.118 (0.320,2.357) | 4.39 E-01 |
| Stage 2 vs. Stage 3 | 125 vs. 85 | 1.288(0.325,3.437) vs. 0.713 (0.218,2.781) | 8.24 E-01 |
| Stage 2 vs. Stage 4 | 125 vs. 28 | 1.288(0.325,3.437) vs.0.713 (0.218,2.781) | 7.42 E-01 |
| Stage 3 vs. Stage 4 | 85 vs. 28 | 0.713 (0.218,2.781) vs. 0.713 (0.218,2.781) | 4.21 E-01 |
| Tissue subtype |  |  |  |
| NOS vs. Clear Cell | 320 vs. 2 | 0.984 (0.281,2.787) vs. 0.559(0.532,0.586) | <1E-12^***^ |
| NOS vs. Solid pattern Predominant | 320 vs. 5 | 0.984 (0.281,2.787) vs. 0.355 (0.150,1.288) | 4.61 E-04^***^ |
| NOS vs. Acinar | 320 vs. 18 | 0.984 (0.281,2.787) vs. 3.848 (0.756,8.067) | 3.41 E-02^*^ |
| Mixed vs. Clear Cell | 107 vs. 2 | 1.870 (0.395,3.666) vs. 0.559(0.532,0.586) | 2.80 E-11^***^ |
| Mixed vs. Solid pattern Predominant | 107 vs. 5 | 1.870 (0.395,3.666) vs. 0.355 (0.15,1.288) | 1.02 E-04^***^ |
| Mixed vs. Acinar | 107 vs. 18 | 1.870 (0.395,3.666) vs. 83.848 (0.756,8.067) | 3.25E-02^*^ |
| Mixed vs. Mucinous | 107 vs. 5 | 1.870 (0.395,3.666) vs. 7.571(6.62,8.522 ) | 4.18E-02^*^ |
| Clear Cell vs. LBC NonMucinous | 2 vs. 19 | 0.559(0.532,0.586) vs. 1.047 (1.909,5.544) | 2.02E-05^***^ |
| Clear Cell vs. Acinar | 2 vs. 18 | 0.559(0.532,0.586) vs. 3.848 (0.756,8.067) | 6.04E-04^***^ |
| Clear Cell vs. Papillary | 2 vs. 23 | 0.559(0.532,0.586) vs. 0.845(0.39,4.142) | 4.86E-03^***^ |
| LBC NonMucinous vs. Solid Pattern Predominant | 19 vs.5 | 1.047 (1.909,5.544) vs. 0.355 (0.15,1.288) | 7.50E-05^***^ |
| Solid Pattern Predominant vs. Acinar | 5 vs. 18 | 0.355 (0.15,1.288) vs. 3.848 (0.756,8.067) | 9.18E-04^***^ |
| Solid Pattern Predominant vs. Papillary | 5 vs. 23 | 0.355 (0.15,1.288) vs. 0.845(0.39,4.142) | 4.76E-02^*^ |
| Mucinous carcinoma vs. Mucinous | 10 vs. 2 | 2.249 (1.007,2.841) vs. 7.571(6.62,8.522) | 9.74E-03^**^ |
| Nodal metastasis status |  |  |  |
| N0 vs. N2 | 331 vs. 74 | 1.597 (0.440,3.800) vs. 0.73 (0.215,2.758) | 3.84E-03^**^ |
| TP53 mutation status |  |  |  |
| Mutation vs. Non-mutation | 223 vs. 279 | 0.592 (0.212,1.631 vs. 2.108 (0.738,4.109) | 2.54E-03^**^ |

MECOM,MDS1 and EVI1 complex locus, also known as PRDM3; PRDM16, PR domain containing 16, also called MEL1;LUAD, lung adenocarcinoma; Reformed Smoker1, <15 years；Reformed Smoker1, >15 years; NOS, lung adenocarcinoma-not otherwise specified; LBC NonMucinous, Lung Bronchioloavleolar Noncarcinmoma; LBC Mucinous, Lung Bronchioloavleolar Carcinoma Mucinous; Papillary, lung papillary adenocarcinoma; Mucinous, mucinous (colloid) carcinoma; Clear cell, Lung clear cell Adenocarcinoma; Mucinous, lung mucinous adenocarcinoma; Mixed, lung adenocarcinoma mixed subtype. ^***^*P*<0.001, ^**^*P*<0.01, ^*^*P*<0.05.
